# An international survey-based assessment of minimally invasive mitral valve surgery

**DOI:** 10.1093/icvts/ivad154

**Published:** 2023-09-15

**Authors:** Ali Fatehi Hassanabad, Umar Imran Hamid, Peyman Sardari Nia

**Affiliations:** Section of Cardiac Surgery, Department of Cardiac Sciences, Libin Cardiovascular Institute, University of Calgary, Calgary, Canada; Department of Cardiothoracic Surgery, Maastricht University Medical Center, Maastricht, Netherlands; Department of Cardiac Surgery, Nottingham University Hospital, Nottingham, UK; Department of Cardiothoracic Surgery, Maastricht University Medical Center, Maastricht, Netherlands

**Keywords:** Minimally invasive cardiac surgery, Minimally invasive mitral valve surgery, International survey

## Abstract

**OBJECTIVES:**

Minimally invasive mitral valve surgery (MIMVS) has been shown to be safe and feasible however its adoption has lagged globally. The international consortium is lacking a set of guidelines that are specific to MIMVS. The aim of this study was to capture the practices of MIMVS in different centres.

**METHODS:**

A survey was constructed containing 52 multiple-choice and open-ended questions about various aspects of MIMVS. The survey was sent to centres that routinely and frequently perform MIMVS. All surgeons provided informed consent for participating in the survey and publication of data.

**RESULTS:**

The survey was sent to 75 known surgeons from whom 32 (42%) completed the survey. All survey responders performed >25 MIMVS cases annually. Twenty (68%) of the surgeons thought that simulation training, MIMVS fellowship and proctorship are all essential prior to commencing an MIMVS program. Eleven (34%) of the surgeons stated that 50–100 MIMVS cases are required to overcome the learning curve, followed by 6 (18%) who said 21–30 cases should suffice. Eighteen (62%) of the surgeons had adopted a fully endoscopic approach for their MIMVS, followed by 15 (51%) surgeons who had performed cases via endoscopic-assisted strategies, 5 (17%) surgeons had conducted the operation under direct visualization and 6 (20%) surgeons had used a robot for their MIMVS.

**CONCLUSIONS:**

The study highlights a marked variability on training and approach to MIMVS. Consensus guidelines should be established to allow standardization of MIMVS.

**Figure:**
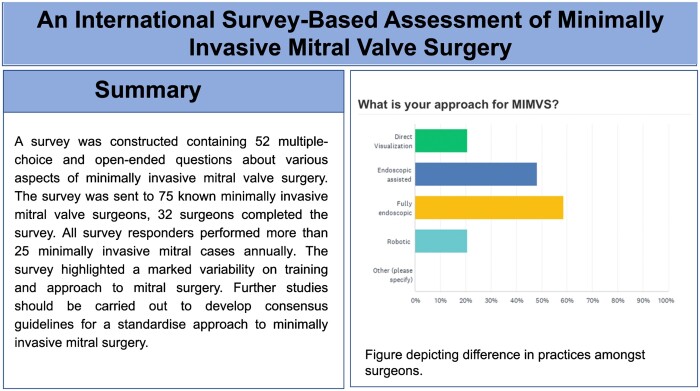

## INTRODUCTION

Minimally invasive cardiac surgery (MICS) has continued to grow and evolve since its introduction in the mid-1990s. These approaches include minimally access sternum-sparing cardiac surgery and can be applied to coronary, valvular and proximal aortic surgery. In addition to being safe and feasible, MICS has been found to confer many benefits, including shorter length of hospital stay, less bleeding and less pain [1–10]. MICS can also be considered for high-risk surgical patients, including the elderly, frail and reoperative patients [11–13]. Furthermore, these clinical advantages can lessen some of the financial burden that is experienced by our healthcare systems [14].

Minimally invasive mitral valve surgery (MIMVS) is a general terminology used to designate all the minimally access approaches applied in addressing atrioventricular valvular pathologies. There is a plethora of techniques and approaches designated as minimally invasive from mini-thoracotomy with direct vision, 2D endoscopic-assisted mini-thoracotomy, robotic-assisted approaches to fully endoscopic with 2D or 3D vision. Although more studies are needed, recent meta-analyses and large registries have found that, compared to sternotomy-based approaches, MIMVS can be associated with the same favourable outcomes [15–18]. Nevertheless, the adoption of MIMVS has not been universal, while centres that have established MIMVS programs appear to have different approaches and preferences. Further standardization, centralization, structural training and specific guidelines for the novices are necessary in helping the adoption of MIMVS. To gain an appreciation of the challenges facing MIMVS and better understand the practical variations that exist among MIMVS surgeons, herein, we have conducted an international survey of surgeons who regularly perform MIMVS. The results of this study provide important insight into the current state of MIMVS at large centres of excellence, and these findings can be used to inform future clinical studies.

## METHODS

This study is based on a survey that was sent to centres around the world that are known to perform MIMVS (Supplementary Material, Appendix SA). The survey (Supplementary Material, Appendix SB), which contained 52 multiple-choice and open-ended questions, was sent via e-mail (Supplementary Material, Appendix SC) to 75 surgeons who perform MIMVS. Centres and surgeons were identified based on their reputation of performing a high volume of MIMVS cases in their region. Responders all provided informed consent for participating in the survey and were given 2 weeks to complete the survey, where a reminder email was sent after the first week. Survey Monkey was used to facilitate the completion of the survey. Therefore, answers remained anonymous. The participants were allowed to pick multiple answers for each question. Responses to the multiple-choice questions were tabulated and presented as bar graphs, while open-ended answers were summarized in tables. The survey consisted of questions pertaining to MIMVS definitions; indications and contraindications for MIMVS; learning curve and training required for MIMVS; handling concomitant diseases; patient selection; and intraoperative considerations.

Statistical analysis of the survey consisted of tabulating all answers; determining percentages of responses for each question; and preparing bar graphs and tables that summarize the results. Since no comparative analysis was done with respect to non-MIMVS for this survey, we did not assess for significance or regression.

### Ethical statement

Ethical approval is not required for the current study as it does not fall within the scope of the medical scientific research with the Human Subjects Act.

## RESULTS

### Participant demographics

**Figure 1:**
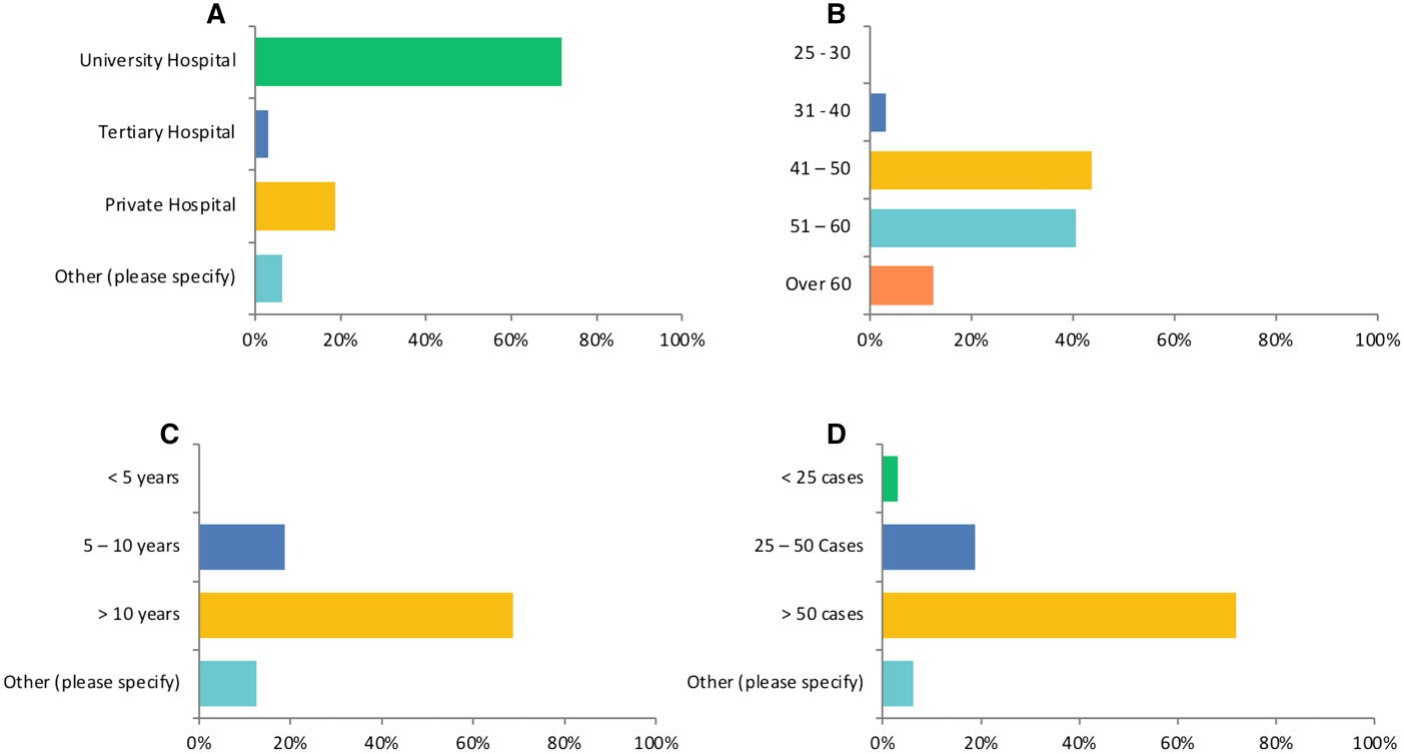
Demographics of survey responders. (**A**) Survey responders’ type of practice. (**B**) Age group of survey responders. (**C**) Number of years of practice of survey responders. (**D**) Number of minimally invasive mitral valve surgery cases performed by the survey responders annually.

The survey was distributed among 75 surgeons who perform MIMVS, from whom 32 (42%) recipients completed the questions. Participants were attending surgeons who perform MIMVS at centres in Europe, North America and Asia. Twenty-three (71%) surgeons practiced in university hospitals, followed by 6 (18%) who worked in the private sector (Fig. 1A). Fourteen (43%) surgeons were between 41 and 50 years old, 13 (40%) were 51–60 years old, 4 (12%) were over 60 years old and 1 (3%) was 31–40 years old (Fig. 1B). Twenty-two (68%) surgeons had been in practice more than 10 years, followed by 6 (18%) who had been in practice for 5–10 years (Fig. 1C). Furthermore, 23 (71%) of the surgeons performed more than 50 MIMVS cases each year, while 6 (18%) conducted 25–50 cases annually (Fig. 1D). Thus, all survey responders performed >25 MIMVS cases annually.

### Learning curve and maintaining expertise

**Figure 2:**
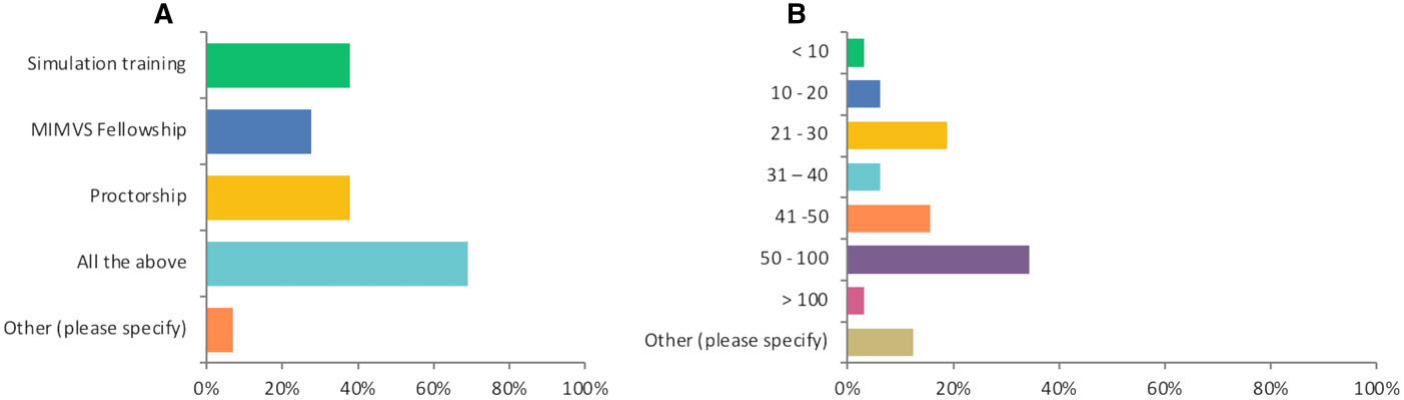
Training and learning curve for MIMVS. (**A**) Type of training required as per survey responders prior to performing MIMVS. (**B**) The learning curve of MIMVS training as per survey responders. MIMVS: minimally invasive mitral valve surgery.

Twenty (68%) of the surgeons thought that simulation training, MIMVS fellowship and proctorship are all essential prior to commencing a MIMVS program (Fig. 2A). Eleven (34%) of the surgeons stated that 50–100 MIMVS cases are required to overcome the learning curve, followed by 6 (18%) who said 21–30 cases should suffice (Fig. 2B). Furthermore, 18 (56%) of the surgeons thought that 2 MIMVS cases per week are needed to maintain the skillset. Moreover, 87.5% of surgeons stated that they benefitted from a dedicated MIMVS program at their centre. Twenty-five (86%) of surgeons believed that novice surgeons should establish their MIMVS practice initially by conducting the operation under direct visualization and gradually transition to endoscopic approaches. Finally, 30 (93%) of surgeons recommended that beginners should aim to avoid re-do cases when starting their MIMVS practices.

### Minimally invasive mitral valve surgery patient selection: indications and contraindications

Eighteen (62%) of the surgeons maintained that the surgeon should decide which patient would be suitable for MIMVS, whereas only 9 (31%) or surveyors believed the heart team and patient should also be a part of the decision-making algorithm. Half of the surgeons said there were no specific institutional guidelines for determining patient suitability for MIMVS. Although 30 of the surgeons (93%) used risk stratification tools; EuroSCORE 14 surgeons (43.75%), STS 6 surgeons (18.75%), both 10 surgeons (31.25%); however, 28 (87%) of the surgeons did not apply a specific score cut-off for MIMVS contraindication. Similarly, 29 (90%) of the surgeons did not consider age to be a contraindication to MIMVS, 22 (68.75%) of the surgeons excluded elevated body mass index as a contraindication to MIMVS, 27 (84%) of surgeons did not consider mitral valve endocarditis as a potential MIMVS contraindication and 16 (55%) of the surgeons were comfortable with concomitant procedures. Finally, 19 (65%) of surgeons believed that lung adhesions constitute an indication for conversion to a sternotomy.

**Figure 3:**
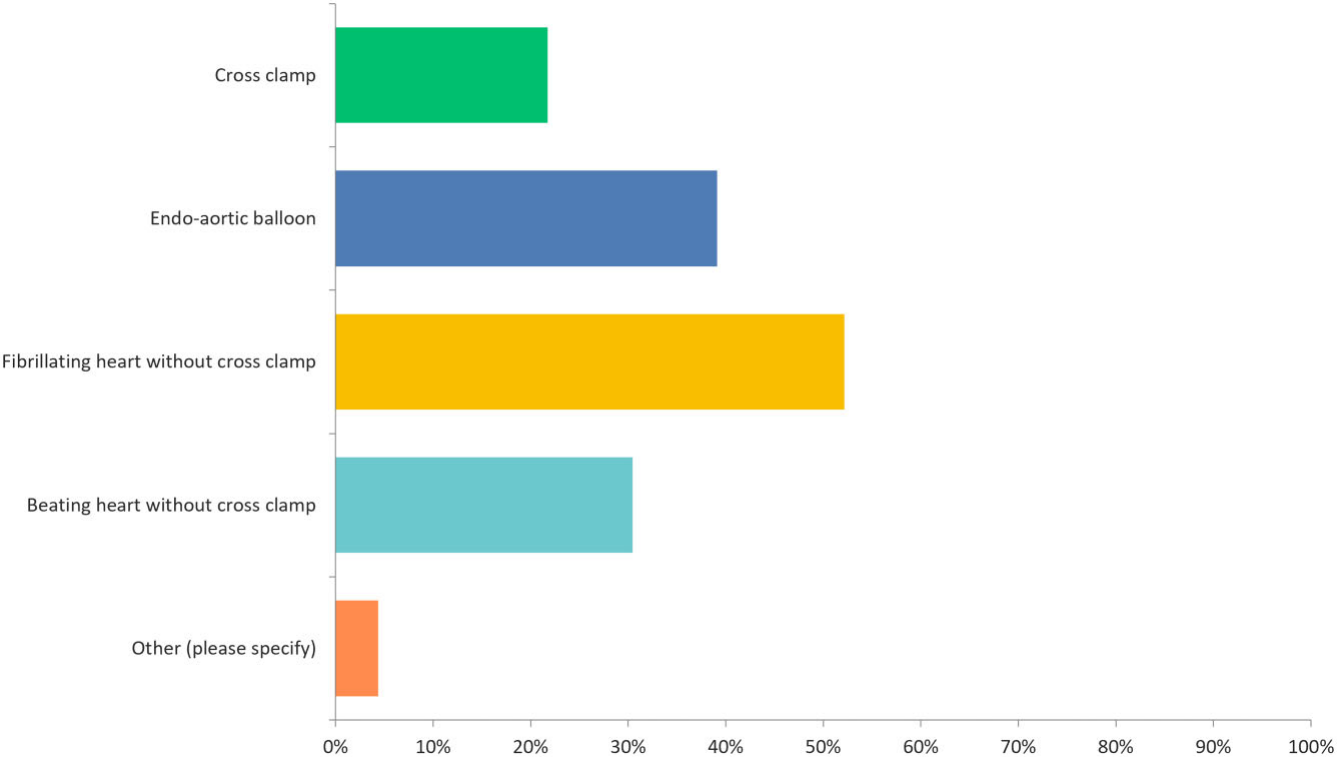
Survey responders’ approach to a porcelain aorta where applying an aortic cross-clamp is not safe or possible.

In contrast, 9 (28%) of the surgeons stated that chest deformity can be considered a contraindication for MIMVS, while 10 (31%) of surgeons indicated that a porcelain aorta should be a contraindication for MIMVS and 8 (25%) surgeons stated that severe peripheral vascular disease can hinder MIMVS. As per survey responders, aortic regurgitation, especially greater than mild aortic regurgitation, can also be considered a contraindication to MIMVS. Although 11 (34%) surgeons did not consider left ventricular ejection fraction to preclude a patient from MIMVS, 13 (40%) surgeons stated that a left ventricular ejection fraction <20% is not suitable for MIMVS. Furthermore, 20 (62%) surgeons indicated that prior thoracic surgery can be used to exclude a patient from undergoing MIMVS, while 11 (34%) surgeons thought that severe mitral annular calcification (MAC) can be prohibitive. Table 1 summarizes the potential contraindications to MIMVS. Among those who indicated that a porcelain aorta should not preclude a patient from MIMVS, majority of the surgeons would conduct the operation with a fibrillating heart without using cross-clamp while others would use an endo-aortic balloon, beating heart without cross-clamp and 5 (21%) surgeons would still consider using a cross-clamp (Fig. 3).

**Table 1:** Summary of potential contraindications to minimally invasive mitral valve surgery

Concomitant procedures
Mitral valve endocarditis
Severe circumferential mitral annular calcification
Previous cardiac surgery
Prior chest radiotherapy
Prior thoracic surgery
Prior thoracic trauma
Prior TAVI
Severe peripheral vascular disease
Porcelain aorta
Aortic valve insufficiency (trace or greater)
BMI >35
Chest wall deformity and/or abnormality (such as pectus excavatum)
Age >65 years old

TAVI: Transcatheter aortic valve implantation; BMI: Body mass index.

### Preoperative and operative considerations

**Figure 4:**
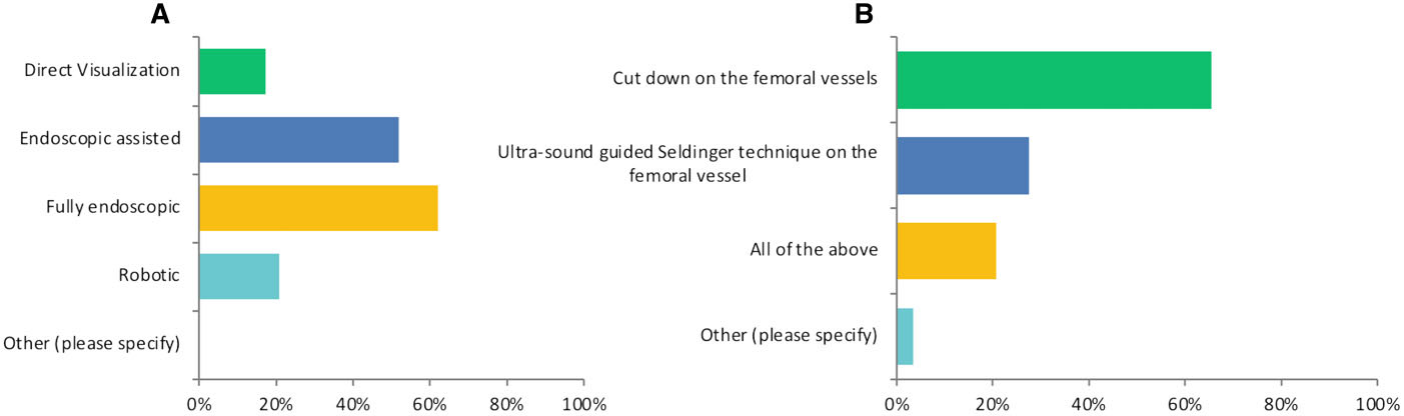
Surgical approach for MIMVS. (**A**) Intraoperative valvular visualization and assessment for MIMVS cases. (**B**) Cannulation strategy for MIMVS. MIMVS: minimally invasive mitral valve surgery.

Twenty-one (72%) of the surgeons said that they obtained a whole-body cat scan (CT) scan preoperatively. Eighteen (62%) of the surgeons had adopted a fully endoscopic approach for their MIMVS, followed by 15 (51%) surgeons who had performed cases via endoscopic-assisted strategies, 5 (17%) surgeons had conducted the operation under direct visualization and 6 (20%) surgeons had used a robot for their MIMVS (Fig. 4A). All surgeons stated that they employed peripheral cannulation, where 19 (65%) surgeons performed a cut-down on the femoral vessels, 8 (27%) surgeons used ultrasound-guided percutaneous techniques and 6 (20%) surgeons had done both (Fig. 4B). Sixteen (55%) of the surgeons used a 2-dimensional camera and 15 surgeons had also used a 3-dimensional camera. Del Nido (15 surgeons, 51%) and Custodial (11 surgeons, 37%) were the most commonly used cardioplegia solutions. Myocardial protection and de-airing strategies are summarized in Tables 2 and 3, respectively.

**Table 2:** Strategies for myocardial protection during minimally invasive mitral valve surgery

Antegrade
Retrograde
Direct ostial
Mild hypothermia

**Table 3:** De-airing strategies for minimally invasive mitral valve surgery cases

Same as sternotomy-based mitral valve surgery
Endo-balloon
Reverse Trendelenburg
Root and LV vent
CO_2_ insufflation

LV: Left ventricle.

### Mitral valve pathologies and repair strategies

**Figure 5:**
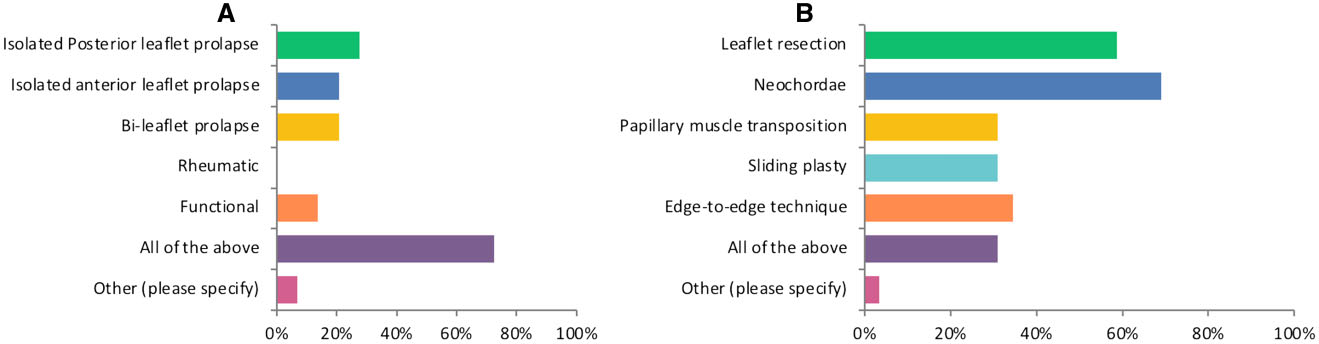
Mitral valve pathologies and repair strategies for MIMVS. (**A**) Valve pathologies amenable to MIMVS. (**B**) Repair strategies for MIMVS. MIMVS: minimally invasive mitral valve surgery.

Twenty-one (72%) surgeons stated that isolated posterior leaflet prolapse, isolated anterior leaflet prolapse, bi-leaflet prolapse, rheumatic and functional mitral regurgitation are all amenable to repair via MIMVS (Fig. 5A). Interestingly, 20 (68%) of the surgeons used neochordae to repair the mitral valve, followed by 17 (58%) surgeons who also employed leaflet resection (Fig. 5B). In addition to these repair strategies, 9 (31%) of the surgeons were comfortable with performing papillary muscle transposition, sliding plasty and edge-to-edge repair. The most commonly used repair technique was neochordae, 26 (89%) of the surgeons used it for their MIMVS. Furthermore, 22 (79.31%) of the surgeons used a complete ring for annuloplasty during MIMVS. Only 3 (10%) of the surgeons did not exclude left atrial appendage in patients with chronic atrial fibrillation who were undergoing MIMVS, where the majority were comfortable with placing sutures from inside or applying a clip through the transverse sinus.

## DISCUSSION

MICS will continue to play an important role in our management of patients with cardiovascular diseases. MICS strategies can be applied to mitral valve pathologies, where MIMVS should be more routinely performed globally in the next 5–10 years. Although MICS can yield favourable outcomes, in some centres, MIMVS can be associated with a longer intraoperative time and a steep learning curve [19–23]. There is real-world evidence supporting the safety of MIMVS when compared to conventional sternum-based mitral valve operations. Given the current state of MIMVS, in this study, we sought to better understand general perceptions surrounding MIMVS by conducting an international survey.

Although subjective, this preliminary work is intended to be based on an international survey that was distributed amongst MIMVS surgeons. Our findings suggest that MIMVS is a multi-faceted area of cardiac surgery that has many nuances, which can be extrapolated to derive consensus statements. Indeed, our findings highlight that, as a community, there is enough collective expertise to consider developing a consensus statement on specific components of MIMVS. For instance, given that most surgeons shared the same opinion on relative indications and potential contraindications for MIMVS, a consensus statement can include those features. For example, surveyors indicated that MIMVS can even be employed in select reoperative cases, which has been shown to be safe in the literature [24]. Moreover, similar to a recent systematic review [25], survey responders stated that MIMVS outcomes can rely on the volume of cases.

The rate of mitral valve repair through MIMVS was also high among survey responders, so the consensus statement should be inclusive of such a finding. The same can be applied to cannulation strategies and having no age cut-off for MIMVS patients. On the other hand, there was a lack of consensus on the use of a particular type of camera, ring and cardioplegia solution. The presence of severe circumferential MAC in MIMVS cases is also contentious, so it should be highlighted. These findings are important since they offer new and important information that can be used by novice and experienced MIMVS surgeons. By developing a consensus statement, indications/contraindications and preoperative investigations can be standardized; operative techniques and considerations can be defined in detail; and postoperative care and follow-up can be outlined. Collectively, these elements should be contextualized by contrasting the role and impact MIMVS can have compared to emerging transcatheter approaches for mitral valve pathologies. Standardizing the applicability of MIMVS can be important in complementing conventional mitral valve surgeries and transcatheter strategies.

Recent retrospective analysis of prospective registry of all-comers for reoperative mitral valve surgery showed excellent comparable results for both minimally invasive as well as for sternotomy. However, the mean EuroSCORE I of whole population was around 12% compared with 30-day mortality of 3.1% of the whole group, indicating that conventional risk scoring systems are inadequate in predicting postoperative mortality in mitral valve surgery [24]. In another study, Bonaros *et al.* found that, in minimally invasive mitral surgery for degenerative disease, chordal replacement yields higher rates of periprocedural success than leaflet resection. Posterior leaflet pathology is an independent predictor of reoperation-free survival [26]. Finally, Berretta *et al.* [27] assessed the operative results of minimally invasive mitral valve operations across different patient risk profiles and evaluated the value of EuroSCORE II predicted risk of mortality model for risk prediction, in the large cohort of Mini-Mitral International Registry. They found that operative results were excellent in low-, intermediate- and high-risk patients but were less satisfactory in extreme risk. Importantly, the EuroSCORE II model overestimated the in-hospital mortality [27]. Collectively, these studies underline that the use of risk scores as current risk-stratifying tools should be used judiciously before excluding patients from MIMVS.

Our survey has also provided insight into the components of the learning curve that exists for MIMVS. Although training MIMVS surgeons globally may be challenging, surgeons who are interested in learning and performing MIMVS can benefit from our survey by pursuing dedicated fellowships and enrolling in proctorship programs. Finally, our study has identified areas that can be further explored in clinical studies. For example, clinical outcomes of MIMVS in patients with severe MAC or those with porcelain aortas can be assessed in large, multicentre studies. Similarly, more clinical data with long follow-up periods are needed to better understand how various repair strategies can influence outcomes.

### Limitations

Although our study benefits from an international group of participants, this survey does have a few limitations. First, as is the case with any survey, response rate can be a limiting factor in generalizing responses. Second, the survey was distributed to only 75 practicing surgeons. Although this constituted a group of surgeons who regularly perform MIMVS, future surveys should aim to include a larger number of participants. Third, and importantly, in this preliminary work, we have not surveyed surgeons who carry out conventional mitral valve surgery and, hence, are missing a control group. We are currently developing a larger survey that will be distributed to MIMVS and conventional mitral valve surgeons. Finally, given the number of participants, we have not stratified participants based on region, gender or age; all of which may affect opinions and approaches to MIMVS. Future surveys should consider these important parameters and determine whether practices vary based on participants’ demographics.

## CONCLUSION

MIMVS is a growing area within cardiac surgery. There is mounting evidence supporting the safety and feasibility of MIMVS. Given the limitations of transcatheter approaches in treating mitral valve diseases, MIMVS can be offered to many patients suffering from various mitral valve pathologies. This study highlights the important considerations of MIMVS, where findings can be used to draft consensus statements that can be used to guide practice.

## Supplementary Material

ivad154_Supplementary_Data

## Data Availability

The authors confirm that the data presented in this manuscript are contained herein.

## ABBREVIATIONS

MAC: Mitral annular calcification
MICS: Minimally invasive cardiac surgery
MIMVS: Minimally invasive mitral valve surgery
