# Supplementary material for: An international survey-based assessment of minimally invasive mitral valve surgery
Source: Interdiscip Cardiovasc Thorac Surg. 2023 Sep 15;37(4):ivad154. doi: 10.1093/icvts/ivad154 (PMC10550782; doi:10.1093/icvts/ivad154)
Supplement: ivad154_Supplementary_Data [file ivad154_supplementary_data.docx]

**Supplementary Material**

Appendix A: Centres that responded to the survey

1 Philadelphia, Pennsylvania, USA

2 Ancona, Italy

3 London UK

4 Seoul, South Korea

5 Turin, Italy

6 Liverpool, UK

7 Medellin, Colombia

8 New Delhi, India

9 Blackpool, UK

10 Singapore

11 Cincinnati, Ohio, USA

12 Calgary, Canada

13 San Francisco, USA

14 Rome, Italy

15 Paris, France

16 Bonn, Germany

17 Massa, Italy

18 London, Ontario, Canada

19 Passau, Germany

20 St Antonius Hospital, Netherlands

21 Montréal, Canada

22 Milan, Italy

23 Nagoya, Japan

24 Innsbruck, Austria

25 Rochester, NY, USA

26 Morgantown, West Virginia, USA

27 Hamburg, Germany

Appendix B: Survey Questions

Q1: I consent to the following survey

Q2: Do you perform MIMVS?

Q3: Please state your Full Name

Q4: Please state the city and country your current hospital is based at

Q5: Which Health sector do you practice MIMVS?

Q6: Please indicate your age group

Q7: How many MIMVS do you perform each year?

Q8: Please indicate the number of years practicing MIMVS?

Q9: What is your repair rate in degenerative mitral valve surgery using MIMVS?

Q10: In your opinion what is the number of cases required to overcome the learning curve for MIMVS?

Q11: To maintain skills for MIMVS how many cases should be performed each week?

Q12: What training should be required prior to commencing a MIMVS program?

Q13: Who should decide the suitability of patient for MIMVS approach?

Q14: Do you have a dedicated team for MIMVS?

Q15: Are there specific institutional guidelines for suitability for MIMVS?

Q16: Do all patients undergoing MIMVS undergo a pre-operative CT scan?

Q17: What is your preferred pre-operative risk evaluation tool for MIMVS?

Q18: Is a specific EuroScore or STS score a contra-indication for MIMVS?

Q19: What is your approach for MIMVS?

Q20: What is your cannulation strategy?

Q21: For peripheral cannulation, what is your access method of choice?

Q22: Which camera do you use?

Q23: Which cardioplegia solution do you prefer for your MIMVS?

Q24: Which mitral valve pathology in your practice are suitable for repair using MIMVS?

Q25: Which techniques do you use to repair the mitral valve during MIMVS?

Q26: Which techniques do you use most often to repair the mitral valve during MIMVS?

Q27: Which annuloplasty technique do you use most often to repair the mitral valve during MIMVS?

Q28: Do you carry out left atrial appendage exclusion in chronic AF patients during MIMVS?

Q29: Do you consider a certain age to be a contraindication for MIMVS?

Q30: If yes, what is your age cut-off?

Q31: Do you consider chest deformity a contraindication for MIMVS?

Q32: If yes, please state which chest deformity would preclude a patient from MIMVS?

Q33: In your practice is a specific BMI contraindication to MIMVS?

Q34: In your practice what degree of aortic regurgitation is considered a contraindication for MIMVS?

Q35: What is your strategy for myocardial protection in the setting of AR and MIMVS? Please state.
Q36: Does ascending aortic calcification preclude a patient from MIMVS?

Q37: If no, what technique do your use to perform the MIMVS?

Q38: Is severe peripheral vascular disease a contraindication for MIMVS?

Q39: If no, what cannulation strategy do you use? Please state.

Q40: What degree of LV impairment is a contraindication to MIMVS?

Q41: Which previous surgery or intervention would preclude a patient for MIMVS?
Q42: Do you consider mitral annular calcification (MAC) as a contraindication to MIMVS?

Q43: If no, what is your approach to MAC in MIMVS?

Q44: Do you consider mitral valve endocarditis as a contraindication to MIMVS?

Q45: Which of the following concomitant procedures in your practice are contraindication to MIMVS?

Q46: What are the indications for conversion to a full sternotomy in your MIMVS practice?

Q47: In your opinion, surgeons in the early stage of MIMVS encountering the above complexities, should they convert to a full sternotomy early?

Q48: What is your deairing strategy for MIMVS?

Q49: For those starting with MIMVS, what would you recommend regarding the conditioning?

Q50: For those starting with MIMVS, what would you recommend regarding the approach?

Q51: For those starting with MIMVS, which of the following patients’ categories should be excluded

Q52: Any suggestions to improve future surveys?

Appendix C: Cover Letter that was sent to the survey responders with the survey attached.

Dear esteemed colleagues,

We would like to invite you to participate in a “Minimally Invasive Mitral Valve Surgery Survey”.

As you know, globally, minimally invasive cardiac surgery (MICS) is continuing to play an important role in the management of patients presenting with various cardiovascular pathologies. Minimally invasive mitral valve surgery (MIMVS) is an area that has gained significant attention over the past decade. It is an approach that can provide excellent clinical outcomes. There are, however, barriers that have prevented the adoption of MIMVS in different regions worldwide. To best identify practice patterns and experiences, we have prepared a comprehensive survey that has been distributed to centres and surgeons who regularly practice MIMVS.

The survey takes approximately 15 minutes to complete. We anticipate that the data analysed from this survey will help to develop standard guidelines on MIMVS and aid us in identifying potential barriers that may exist in your region and/or practice in further expanding your MIMVS program.

At the end of the survey, a free text box will be available for any comments about the survey or suggestions to improve future surveys.

If you would like to participate, follow this link to the Survey:

Take the Survey

Or copy and paste the URL below into your internet browser:

https://www.surveymonkey.com/r/23QXH8Y

Consent: Providing electronic consent will be accepted as a proxy for signing a consent form, a copy of which can be downloaded here or from the first page of the survey.

You can save your responses, then close the browser and return to the survey using the same link up to one week later. The survey is compatible with mobile devices.

Where can I get further information?

If you would like to discuss the study, please do not hesitate to contact Dr. Sardari Nia (peyman.sardarinia@mumc.nl; +31-(0)-43-3877095)

A participant information sheet providing further information about the survey can be downloaded here.

We thank you for your time and expertise.

Dr. Peyman Sardari Nia

# Maastricht University Medical Center (MUMC+), The Netherlands

# Appendix C: all the survey answers.

# Q1 I consent to the following:

Answered: 32 Skipped: 0

(no label)

Taking part in an online...

The data that I provide...

0% 10% 20% 30% 40% 50% 60% 70% 80% 90% 100%


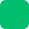

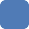
Yes No

| (no label) | **YES** | **NO** | **TOTAL** |
| --- | --- | --- | --- |
| Taking part in an online survey | 100.00% | 0.00% |  |
|  | 32 | 0 | 32 |

The data that I provide during this study will be used for publications. The data may be used in future research projects (with appropriate ethics approval and de-identification)

100.00%

32

0.00%

0 32

# Q2 Do you perform MIMVS?

Answered: 32 Skipped: 0

Yes

|  |  |  |  |  |  |  |  |  |  |
| --- | --- | --- | --- | --- | --- | --- | --- | --- | --- |
|  | | | | | | | | | |
|  |  |  |  |  |  |  |  |  |  |

No

0% 10% 20% 30% 40% 50% 60% 70% 80% 90% 100%

| **ANSWER CHOICES RESPONSES** | |
| --- | --- |
| Yes | 100.00% 32 |

No 0.00% 0

TOTAL 32

# Q5 Which Health sector do you practice MIMVS?

Answered: 32 Skipped: 0

University Hospital

|  | | | | | | | |  |  |
| --- | --- | --- | --- | --- | --- | --- | --- | --- | --- |
|  | |  |  |  |  |  |  |  |  |
|  |  |  |  |  |  |  |  |  |  |

Tertiary Hospital

Private Hospital

Other (please

specify)

0% 10% 20% 30% 40% 50% 60% 70% 80% 90% 100%

| **ANSWER CHOICES** | **RESPONSES** | |
| --- | --- | --- |
| University Hospital | 71.88% | 23 |
| Tertiary Hospital | 3.13% | 1 |
| Private Hospital | 18.75% | 6 |

Other (please specify)

6.25% 2

TOTAL 32

| **#** | **OTHER (PLEASE SPECIFY)** | **DATE** |
| --- | --- | --- |
| 1 | University and private hospital | 3/28/2022 10:01 AM |

2 Teaching hospital 3/24/2022 1:40 PM

# Q6 Please indicate your age group:

Answered: 32 Skipped: 0

25 - 30

|  | | | | |  |  |  |  |  |
| --- | --- | --- | --- | --- | --- | --- | --- | --- | --- |
|  |  |  |  |  |  |  |  |  |  |
|  | | | |  |  |  |  |  |  |
|  |  |  |  |  |  |  |  |  |  |
|  | |  |  |  |  |  |  |  |  |

31 - 40

41 – 50

51 – 60

Over 60

0% 10% 20% 30% 40% 50% 60% 70% 80% 90% 100%

| **ANSWER CHOICES** | **RESPONSES** | |
| --- | --- | --- |
| 25 - 30 | 0.00% | 0 |
| 31 - 40 | 3.13% | 1 |
| 41 – 50 | 43.75% | 14 |
| 51 – 60 | 40.63% | 13 |

Over 60

12.50% 4

TOTAL 32

# Q7 How many MIMVS do you perform each year?

Answered: 32 Skipped: 0

< 25 cases

25 – 50 Cases

> 50 cases

Other (please

specify)

0% 10% 20% 30% 40% 50% 60% 70% 80% 90% 100%

| **ANSWER CHOICES** | **RESPONSES** | |
| --- | --- | --- |
| < 25 cases | 3.13% | 1 |
| 25 – 50 Cases | 18.75% | 6 |
| > 50 cases | 71.88% | 23 |

Other (please specify)

6.25% 2

TOTAL 32

| **#** | **OTHER (PLEASE SPECIFY)** | **DATE** |
| --- | --- | --- |
| 1 | 200+ | 4/14/2022 7:36 AM |

2 100 -150 4/13/2022 5:18 PM

# Q8 Please indicate the number of years practicing MIMVS?

Answered: 32 Skipped: 0

< 5 years

|  | | | | | | |  |  |  |
| --- | --- | --- | --- | --- | --- | --- | --- | --- | --- |
|  | |  |  |  |  |  |  |  |  |
|  |  |  |  |  |  |  |  |  |  |

5 – 10 years

> 10 years

Other (please

specify)

0% 10% 20% 30% 40% 50% 60% 70% 80% 90% 100%

| **ANSWER CHOICES** | **RESPONSES** | |
| --- | --- | --- |
| < 5 years | 0.00% | 0 |
| 5 – 10 years | 18.75% | 6 |
| > 10 years | 68.75% | 22 |

Other (please specify)

12.50% 4

TOTAL 32

| **#** | **OTHER (PLEASE SPECIFY)** | **DATE** |
| --- | --- | --- |
| 1 | 20 | 4/14/2022 7:36 AM |
| 2 | 25 ys | 4/13/2022 5:18 PM |
| 3 | ⁸ | 3/28/2022 10:54 PM |

4 > 20 years 3/28/2022 8:02 AM

# Q9 What is your repair rate in degenerative mitral valve surgery using MIMVS?

Answered: 32 Skipped: 0

Less than 25%

Between 25%-50%

Between 50%-75%

Between 75% -

90%

Between 90 -

95%

> 95%

0% 10% 20% 30% 40% 50% 60% 70% 80% 90% 100%

| **ANSWER CHOICES** | **RESPONSES** | |
| --- | --- | --- |
| Less than 25% | 0.00% | 0 |
| Between 25%-50% | 0.00% | 0 |
| Between 50%-75% | 3.13% | 1 |
| Between 75% - 90% | 12.50% | 4 |
| Between 90 - 95% | 15.63% | 5 |

> 95%

68.75% 22

TOTAL 32

# Q10 In your opinion what is the number of cases required to overcome the learning curve for MIMVS?

Answered: 32 Skipped: 0

< 10

10 - 20

21 - 30

31 – 40

41 -50

50 - 100

> 100

Other (please

specify)

0% 10% 20% 30% 40% 50% 60% 70% 80% 90% 100%

| **ANSWER CHOICES RESPONSES** | | | |
| --- | --- | --- | --- |
| < | 10 | 3.13% | 1 |
| 10 - 20 | | 6.25% | 2 |
| 21 - 30 | | 18.75% | 6 |
| 31 – 40 | | 6.25% | 2 |
| 41 -50 | | 15.63% | 5 |
| 50 - 100 | | 34.38% | 11 |
| > | 100 | 3.13% | 1 |

Other (please specify)

12.50% 4

TOTAL 32

| **#** | **OTHER (PLEASE SPECIFY)** | **DATE** |
| --- | --- | --- |
| 1 | Probably close to 200 | 4/13/2022 2:07 PM |
| 2 | Depends on level of MV surgery experience. 10 for transition to MIS, 50 for more complex pathologies, 100 for endoscopic | 3/29/2022 10:42 AM |

3 Depends on prior experience with mitral repair and endoscope, but generally feel that with some prior experience, mentorship and proctorship, learning curve can be as short as 20-30 cases, but can be as long as 100 cases

3/25/2022 1:36 PM

4 >50 3/24/2022 1:40 PM

# Q11 To maintain skills for MIMVS how many cases should be performed each week?

Answered: 32 Skipped: 0

1

|  | | | | | |  |  |  |  |
| --- | --- | --- | --- | --- | --- | --- | --- | --- | --- |
|  |  |  |  |  |  |  |  |  |  |
|  |  |  |  |  |  |  |  |  |  |
|  |  |  |  |  |  |  |  |  |  |

2

3

4

5

6

Other (please

specify)

0% 10% 20% 30% 40% 50% 60% 70% 80% 90% 100%

| **ANSWER CHOICES RESPONSES** | |
| --- | --- |
| 1 | 28.13% 9 |
| 2 | 56.25% 18 |
| 3 | 9.38% 3 |
| 4 | 0.00% 0 |
| 5 | 0.00% 0 |
| 6 | 0.00% 0 |

Other (please specify)

6.25% 2

TOTAL 32

| **#** | **OTHER (PLEASE SPECIFY)** | **DATE** |
| --- | --- | --- |
| 1 | for experienced mini access surgeons 1/ For more juniors 2 /weeek | 3/28/2022 10:01 AM |

2 2-3 cases per month 3/22/2022 9:11 PM

# Q12 What training should be required prior to commencing a MIMVS program?

Answered: 29 Skipped: 3

Simulation training

MIMVS

Fellowship

Proctorship

All the above

Other (please

specify)

0% 10% 20% 30% 40% 50% 60% 70% 80% 90% 100%

| **ANSWER CHOICES** | **RESPONSES** | |
| --- | --- | --- |
| Simulation training | 37.93% | 11 |
| MIMVS Fellowship | 27.59% | 8 |
| Proctorship | 37.93% | 11 |
| All the above | 68.97% | 20 |

Other (please specify)

6.90% 2

Total Respondents: 29

| **#** | **OTHER (PLEASE SPECIFY)** | **DATE** |
| --- | --- | --- |
| 1 | Have experience with open mitral repair | 3/28/2022 8:02 PM |

2 Mentorship 3/28/2022 10:08 AM

# Q13 Who should decide the suitability of patient for MIMVS approach?

Answered: 29 Skipped: 3

Surgeon

Patient

Heart Team

All the above

Other (please

specify)

0% 10% 20% 30% 40% 50% 60% 70% 80% 90% 100%

| **ANSWER CHOICES** | **RESPONSES** | |
| --- | --- | --- |
| Surgeon | 62.07% | 18 |
| Patient | 6.90% | 2 |
| Heart Team | 17.24% | 5 |
| All the above | 31.03% | 9 |

Other (please specify)

0.00% 0

Total Respondents: 29

**# OTHER (PLEASE SPECIFY) DATE**

There are no responses.

# Q14 Do you have a dedicated team for MIMVS?

Answered: 32 Skipped: 0

Yes

No

0% 10% 20% 30% 40% 50% 60% 70% 80% 90% 100%

| **ANSWER CHOICES RESPONSES** | |
| --- | --- |
| Yes | 87.50% 28 |

No 12.50% 4

TOTAL 32

# Q15 Are there specific institutional guidelines for suitability for MIMVS?

Answered: 32 Skipped: 0

Yes

|  |  |  |  |  |  |  |  |  |  |
| --- | --- | --- | --- | --- | --- | --- | --- | --- | --- |
|  | | | | |  |  |  |  |  |
|  |  |  |  |  |  |  |  |  |  |
|  | | | | |  |  |  |  |  |
|  |  |  |  |  |  |  |  |  |  |

No

Other (please

specify)

0% 10% 20% 30% 40% 50% 60% 70% 80% 90% 100%

| **ANSWER CHOICES** | **RESPONSES** | |
| --- | --- | --- |
| Yes | 50.00% | 16 |
| No | 50.00% | 16 |

Other (please specify)

0.00% 0

TOTAL 32

**# OTHER (PLEASE SPECIFY) DATE**

There are no responses.

# Q16 Do all patients undergoing MIMVS undergo a pre-operative CT scan?

Answered: 29 Skipped: 3

None

|  | | | | | | | |  |  |
| --- | --- | --- | --- | --- | --- | --- | --- | --- | --- |
|  |  |  |  |  |  |  |  |  |  |
|  | |  |  |  |  |  |  |  |  |
|  |  |  |  |  |  |  |  |  |  |

Thorax only

Whole body

Only if there

is an...

Other (please

specify)

0% 10% 20% 30% 40% 50% 60% 70% 80% 90% 100%

| **ANSWER CHOICES** | **RESPONSES** | |
| --- | --- | --- |
| None | 3.45% | 1 |
| Thorax only | 3.45% | 1 |
| Whole body | 72.41% | 21 |
| Only if there is an indication | 20.69% | 6 |

Other (please specify)

6.90% 2

Total Respondents: 29

| **#** | **OTHER (PLEASE SPECIFY)** | **DATE** |
| --- | --- | --- |
| 1 | Case-by-case. Thorax is minimal standard | 3/29/2022 10:42 AM |

2 we can them from proximal carotids to femorals 3/25/2022 1:36 PM

# Q17 What is your preferred pre-operative risk evaluation tool for MIMVS?

Answered: 32 Skipped: 0

Euroscore

|  | | | | |  |  |  |  |  |
| --- | --- | --- | --- | --- | --- | --- | --- | --- | --- |
|  | | | |  |  |  |  |  |  |
|  |  |  |  |  |  |  |  |  |  |

STS

Both

Other (please

specify)

0% 10% 20% 30% 40% 50% 60% 70% 80% 90% 100%

| **ANSWER CHOICES** | **RESPONSES** | |
| --- | --- | --- |
| Euroscore | 43.75% | 14 |
| STS | 18.75% | 6 |
| Both | 31.25% | 10 |

Other (please specify)

6.25% 2

TOTAL 32

| **#** | **OTHER (PLEASE SPECIFY)** | **DATE** |
| --- | --- | --- |
| 1 | Don't really use one...but if I do, I calculate both | 3/25/2022 1:36 PM |

2 clinical evaluation 3/21/2022 1:56 PM

# Q18 Is a specific Euroscore or STS score a contra-indication for MIMVS?

Answered: 32 Skipped: 0

< 5

5 - 10

> 10

None

Other (please

specify)

0% 10% 20% 30% 40% 50% 60% 70% 80% 90% 100%

| **ANSWER CHOICES RESPONSES** | | | |
| --- | --- | --- | --- |
| < | 5 | 3.13% | 1 |
| 5 - 10 | | 0.00% | 0 |
| > | 10 | 3.13% | 1 |
| None | | 87.50% | 28 |

Other (please specify)

6.25% 2

TOTAL 32

| **#** | **OTHER (PLEASE SPECIFY)** | **DATE** |
| --- | --- | --- |
| 1 | Euroscore per se is not a prohibitive factor, but rather the cause of high Euroscore in some cases | 3/29/2022 10:42 AM |

2 Contraindications are related to specific clinical and anatomical conditions 3/22/2022 9:31 AM

# Q19 What is your approach for MIMVS?

Answered: 29 Skipped: 3

Direct Visualization

|  | | | | | | |  |  |  |
| --- | --- | --- | --- | --- | --- | --- | --- | --- | --- |
|  |  |  |  |  |  |  |  |  |  |
|  | |  |  |  |  |  |  |  |  |
|  |  |  |  |  |  |  |  |  |  |

Endoscopic assisted

Fully endoscopic

Robotic

Other (please

specify)

0% 10% 20% 30% 40% 50% 60% 70% 80% 90% 100%

| **ANSWER CHOICES** | **RESPONSES** | |
| --- | --- | --- |
| Direct Visualization | 17.24% | 5 |
| Endoscopic assisted | 51.72% | 15 |
| Fully endoscopic | 62.07% | 18 |
| Robotic | 20.69% | 6 |

Other (please specify)

0.00% 0

Total Respondents: 29

**# OTHER (PLEASE SPECIFY) DATE**

There are no responses.

# Q20 What is your cannulation strategy?

Answered: 29 Skipped: 3

Central

|  |  |  |  |  |  |  |  |  |  |
| --- | --- | --- | --- | --- | --- | --- | --- | --- | --- |
|  | | | | | | | | | |
|  |  |  |  |  |  |  |  |  |  |

Peripheral

Other (please

specify)

0% 10% 20% 30% 40% 50% 60% 70% 80% 90% 100%

| **ANSWER CHOICES** | **RESPONSES** | |
| --- | --- | --- |
| Central | 3.45% | 1 |
| Peripheral | 100.00% | 29 |

Other (please specify)

6.90% 2

Total Respondents: 29

| **#** | **OTHER (PLEASE SPECIFY)** | **DATE** |
| --- | --- | --- |
| 1 | Sometimes Axillary artery | 3/28/2022 10:11 AM |

2 central id controindication for peripheral cannulation 3/22/2022 3:23 PM

# Q21 For peripheral cannulation, what is your access method of choice?

Answered: 29 Skipped: 3

Cut down on the femoral...

|  | | | | | | |  |  |  |
| --- | --- | --- | --- | --- | --- | --- | --- | --- | --- |
|  | | |  |  |  |  |  |  |  |
|  |  |  |  |  |  |  |  |  |  |
|  | |  |  |  |  |  |  |  |  |
|  |  |  |  |  |  |  |  |  |  |

Ultra-sound

guided...

All of the

above

Other (please

specify)

0% 10% 20% 30% 40% 50% 60% 70% 80% 90% 100%

| **ANSWER CHOICES** | **RESPONSES** | |
| --- | --- | --- |
| Cut down on the femoral vessels | 65.52% | 19 |
| Ultra-sound guided Seldinger technique on the femoral vessel | 27.59% | 8 |
| All of the above | 20.69% | 6 |

Other (please specify)

3.45% 1

Total Respondents: 29

| **#** | **OTHER (PLEASE SPECIFY)** | **DATE** |
| --- | --- | --- |
| 1 | Over 90% cutdown | 3/29/2022 10:42 AM |

# Q22 Which camera do you use?

Answered: 29 Skipped: 3

2D camera

3D camera

Other (please

specify)

0% 10% 20% 30% 40% 50% 60% 70% 80% 90% 100%

| **ANSWER CHOICES** | **RESPONSES** | |
| --- | --- | --- |
| 2D camera | 55.17% | 16 |
| 3D camera | 51.72% | 15 |

Other (please specify)

3.45% 1

Total Respondents: 29

| **#** | **OTHER (PLEASE SPECIFY)** | **DATE** |
| --- | --- | --- |
| 1 | Over 80% 2D | 3/29/2022 10:42 AM |

# Q23 Which cardioplegia solution do you prefer for your MIMVS?

Answered: 29 Skipped: 3

Del Nido

|  | | | | | |  |  |  |  |
| --- | --- | --- | --- | --- | --- | --- | --- | --- | --- |
|  | | | |  |  |  |  |  |  |
|  |  |  |  |  |  |  |  |  |  |

Blood

Custodial

Other (please

specify)

0% 10% 20% 30% 40% 50% 60% 70% 80% 90% 100%

| **ANSWER CHOICES** | **RESPONSES** | |
| --- | --- | --- |
| Del Nido | 51.72% | 15 |
| Blood | 31.03% | 9 |
| Custodial | 37.93% | 11 |

Other (please specify)

6.90% 2

Total Respondents: 29

| **#** | **OTHER (PLEASE SPECIFY)** | **DATE** |
| --- | --- | --- |
| 1 | St Thomas | 3/28/2022 2:54 PM |

2 St Thomas 3/23/2022 3:54 PM

# Q24 Which mitral valve pathology in your practice are suitable for repair using MIMVS?

Answered: 29 Skipped: 3

Isolated Posterior...

Isolated anterior...

Bi-leaﬂet prolapse

Rheumatic

Functional All of the

above

Other (please

specify)

0% 10% 20% 30% 40% 50% 60% 70% 80% 90% 100%

| **ANSWER CHOICES** | **RESPONSES** | |
| --- | --- | --- |
| Isolated Posterior leaflet prolapse | 27.59% | 8 |
| Isolated anterior leaflet prolapse | 20.69% | 6 |
| Bi-leaflet prolapse | 20.69% | 6 |
| Rheumatic | 0.00% | 0 |
| Functional | 13.79% | 4 |
| All of the above | 72.41% | 21 |

Other (please specify)

6.90% 2

Total Respondents: 29

| **#** | **OTHER (PLEASE SPECIFY)** | **DATE** |
| --- | --- | --- |
| 1 | E | 4/3/2022 11:30 PM |

2 With the exception of calcified anulus or leaflets 3/22/2022 9:31 AM

# Q25 Which techniques do you use to repair the mitral valve during MIMVS?

Answered: 29 Skipped: 3

Leaﬂet resection

|  | | | | | | |  |  |  |
| --- | --- | --- | --- | --- | --- | --- | --- | --- | --- |
|  | | | |  |  |  |  |  |  |
|  |  |  |  |  |  |  |  |  |  |

Neochordae

Papillary muscle...

Sliding plasty

Edge-to-edge technique

All of the

above

Other (please

specify)

0% 10% 20% 30% 40% 50% 60% 70% 80% 90% 100%

| **ANSWER CHOICES** | **RESPONSES** | |
| --- | --- | --- |
| Leaflet resection | 58.62% | 17 |
| Neochordae | 68.97% | 20 |
| Papillary muscle transposition | 31.03% | 9 |
| Sliding plasty | 31.03% | 9 |
| Edge-to-edge technique | 34.48% | 10 |
| All of the above | 31.03% | 9 |

Other (please specify)

3.45% 1

Total Respondents: 29

| **#** | **OTHER (PLEASE SPECIFY)** | **DATE** |
| --- | --- | --- |
| 1 | and many more | 3/29/2022 10:42 AM |

# Q26 Which techniques do you use most often to repair the mitral valve during MIMVS?

Answered: 29 Skipped: 3

Leaﬂet resection

|  | | | |  |  |  |  |  |  |
| --- | --- | --- | --- | --- | --- | --- | --- | --- | --- |
|  |  |  |  |  |  |  |  |  |  |
|  | | | | | | | | |  |
|  |  |  |  |  |  |  |  |  |  |

Neochordae

Papillary muscle...

Sliding plasty

Edge-to-edge technique

All of the

above

Other (please

specify)

0% 10% 20% 30% 40% 50% 60% 70% 80% 90% 100%

| **ANSWER CHOICES** | **RESPONSES** | |
| --- | --- | --- |
| Leaflet resection | 31.03% | 9 |
| Neochordae | 89.66% | 26 |
| Papillary muscle transposition | 0.00% | 0 |
| Sliding plasty | 3.45% | 1 |
| Edge-to-edge technique | 3.45% | 1 |
| All of the above | 3.45% | 1 |

Other (please specify)

0.00% 0

Total Respondents: 29

**# OTHER (PLEASE SPECIFY) DATE**

There are no responses.

# Q27 Which annuloplasty technique do you use most often to repair the mitral valve during MIMVS?

Answered: 29 Skipped: 3

Complete ring

|  | | | | | | | |  |  |
| --- | --- | --- | --- | --- | --- | --- | --- | --- | --- |
|  | | |  |  |  |  |  |  |  |
|  |  |  |  |  |  |  |  |  |  |

A band

Semirigid ring

Other (please

specify)

0% 10% 20% 30% 40% 50% 60% 70% 80% 90% 100%

| **ANSWER CHOICES** | **RESPONSES** | |
| --- | --- | --- |
| Complete ring | 75.86% | 22 |
| A band | 17.24% | 5 |
| Semirigid ring | 24.14% | 7 |

Other (please specify)

3.45% 1

Total Respondents: 29

| **#** | **OTHER (PLEASE SPECIFY)** | **DATE** |
| --- | --- | --- |
| 1 | band with complete ring fashion | 4/14/2022 7:36 AM |

# Q28 Do you carry out left atrial appendage exclusion in chronic AF patients during MIMVS?

Answered: 29 Skipped: 3

Yes, by suturing fro...

|  | | | | | |  |  |  |  |
| --- | --- | --- | --- | --- | --- | --- | --- | --- | --- |
|  | | | | |  |  |  |  |  |
|  |  |  |  |  |  |  |  |  |  |
|  |  |  |  |  |  |  |  |  |  |
|  |  |  |  |  |  |  |  |  |  |
|  |  |  |  |  |  |  |  |  |  |
|  |  |  |  |  |  |  |  |  |  |

Yes, by placing a cl...

No

Other (please

specify)

0% 10% 20% 30% 40% 50% 60% 70% 80% 90% 100%

| **ANSWER CHOICES** | **RESPONSES** | |
| --- | --- | --- |
| Yes, by suturing from inside | 55.17% | 16 |
| Yes, by placing a clip through transverse sinus | 48.28% | 14 |
| No | 10.34% | 3 |

Other (please specify)

10.34% 3

Total Respondents: 29

| **#** | **OTHER (PLEASE SPECIFY)** | **DATE** |
| --- | --- | --- |
| 1 | Cut and sew | 4/15/2022 1:41 PM |
| 2 | Depending on preop scan of the LAA base configuration and CT anatomy | 3/29/2022 10:42 AM |

3 Surgical stapler through transverse sinus 3/22/2022 11:36 AM

# Q29 Do you consider a certain age to be a contraindication for MIMVS?

Answered: 32 Skipped: 0

Yes

|  |  |  |  |  |  |  |  |  |  |
| --- | --- | --- | --- | --- | --- | --- | --- | --- | --- |
|  |  |  |  |  |  |  |  |  |  |
|  |  |  |  |  |  |  |  |  |  |
|  | | | | | | | | |  |
|  |  |  |  |  |  |  |  |  |  |

No

0% 10% 20% 30% 40% 50% 60% 70% 80% 90% 100%

| **ANSWER CHOICES RESPONSES** | |
| --- | --- |
| Yes | 9.38% 3 |

No 90.63% 29

TOTAL 32

# Q30 If yes, what is your age cutoff?

Answered: 5 Skipped: 27

65 – 70

71 – 75

76 – 80

> 80

Other

0% 10% 20% 30% 40% 50% 60% 70% 80% 90% 100%

| **ANSWER CHOICES RESPONSES** | |
| --- | --- |
| 65 – 70 | 20.00% 1 |
| 71 – 75 | 0.00% 0 |
| 76 – 80 | 0.00% 0 |
| > 80 | 40.00% 2 |

Other

40.00% 2

TOTAL 5

# Q31 Do you consider chest deformity a contraindication for MIMVS?

Answered: 32 Skipped: 0

Yes

|  | | |  |  |  |  |  |  |  |
| --- | --- | --- | --- | --- | --- | --- | --- | --- | --- |
|  |  |  |  |  |  |  |  |  |  |
|  | | | | |  |  |  |  |  |
|  |  |  |  |  |  |  |  |  |  |
|  | | |  |  |  |  |  |  |  |
|  |  |  |  |  |  |  |  |  |  |

No

Other (please

specify)

0% 10% 20% 30% 40% 50% 60% 70% 80% 90% 100%

| **ANSWER CHOICES** | **RESPONSES** | |
| --- | --- | --- |
| Yes | 28.13% | 9 |
| No | 50.00% | 16 |

Other (please specify)

21.88% 7

TOTAL 32

| **#** | **OTHER (PLEASE SPECIFY)** | **DATE** |
| --- | --- | --- |
| 1 | Case by case, but rarely | 3/29/2022 10:42 AM |
| 2 | Sometimes. Depends on the CT imaging | 3/28/2022 8:02 PM |
| 3 | Depending from the degree of deformity | 3/28/2022 2:54 PM |
| 4 | depends on the degree | 3/28/2022 10:08 AM |
| 5 | relative...depends on CT | 3/25/2022 1:36 PM |
| 6 | It will depends on the preop CT scan | 3/22/2022 9:11 PM |

7 sometimes 3/21/2022 6:26 PM

# Q32 If yes, please state which chest deformity would preclude a patient from MIMVS?

Answered: 15 Skipped: 17

| **#** | **RESPONSES** | |  | **DATE** |
| --- | --- | --- | --- | --- |
| 1 | Severe Pectus | | | 4/14/2022 3:38 PM |
| 2 | Pectus | | | 4/13/2022 2:10 PM |
| 3 | Pectus | excavatum | | 4/13/2022 2:07 PM |
| 4 | Severe scoliosis with flat or excavated chest, and left sided heart location | | | 3/29/2022 10:42 AM |
| 5 | Pectus Excavatum in some cases, if severe | | | 3/28/2022 8:02 PM |
| 6 | Pectus | excavatum | | 3/28/2022 6:55 PM |
| 7 | All deformity precluding a safe procedure on ct scan | | | 3/28/2022 2:54 PM |
| 8 | Perçue excavatum | | | 3/28/2022 12:25 PM |
| 9 | Pectum Excavatume ; Severe scolyosis | | | 3/28/2022 8:02 AM |
| 10 | None are absolute. Depends on CT | | | 3/25/2022 1:36 PM |
| 11 | pectus carinatum | | | 3/23/2022 3:54 PM |
| 12 | Very defmorative Pectus excavatus | | | 3/22/2022 9:31 AM |
| 13 | bad scoliosis | | | 3/21/2022 6:26 PM |
| 14 | NA | | | 3/21/2022 2:15 PM |

15 any if pronounced 3/21/2022 1:57 PM

# Q33 In your practice is a specific BMI contraindication to MIMVS?

Answered: 32 Skipped: 0

30 – 35

35 – 40

> 40

None

Other (please

specify)

0% 10% 20% 30% 40% 50% 60% 70% 80% 90% 100%

| **ANSWER CHOICES RESPONSES** | | | |
| --- | --- | --- | --- |
| 30 – 35 | | 0.00% | 0 |
| 35 – 40 | | 6.25% | 2 |
| > | 40 | 15.63% | 5 |
| None | | 68.75% | 22 |

Other (please specify)

9.38% 3

TOTAL 32

| **#** | **OTHER (PLEASE SPECIFY)** | **DATE** |
| --- | --- | --- |
| 1 | BMI >42 | 3/28/2022 10:54 PM |
| 2 | Depends on the CT imaging | 3/28/2022 8:02 PM |

3 It will depend on the perop Ct scan 3/22/2022 9:11 PM

# Q34 In your practice what degree of aortic regurgitation is considered a contraindication for MIMVS?

Answered: 29 Skipped: 3

Trace AR

Mild AR

Mod AR

Severe AR

None

Other (please

specify)

0% 10% 20% 30% 40% 50% 60% 70% 80% 90% 100%

| **ANSWER CHOICES** | **RESPONSES** | |
| --- | --- | --- |
| Trace AR | 6.90% | 2 |
| Mild AR | 3.45% | 1 |
| Mod AR | 44.83% | 13 |
| Severe AR | 41.38% | 12 |
| None | 17.24% | 5 |

Other (please specify)

10.34% 3

Total Respondents: 29

| **#** | **OTHER (PLEASE SPECIFY)** | **DATE** |
| --- | --- | --- |
| 1 | We deal with the aortic valve endoscopicaly if required. | 3/30/2022 3:22 PM |
| 2 | In case of relevant AR we adresse the AV also | 3/28/2022 10:11 AM |

3 moderate or greater 3/25/2022 1:36 PM

# Q35 What is your strategy for myocardial protection in the setting of AR and MIMVS? Please state.

Answered: 32 Skipped: 0

| **#** | **RESPONSES** | **DATE** |
| --- | --- | --- |
| 1 | more cardioplegic solution, systemic cooling | 5/2/2022 5:34 AM |
| 2 | percutaneous retrograde | 4/15/2022 4:52 PM |
| 3 | Direct ostia cannulation | 4/15/2022 1:41 PM |
| 4 | Antegrade and retrograde | 4/14/2022 3:38 PM |
| 5 | antegrade cp + retrograde if possible | 4/14/2022 7:36 AM |
| 6 | High flow cardioplegia | 4/13/2022 5:18 PM |
| 7 | If trace or mild AR, cross clamp cold cardiologia via aortic root | 4/13/2022 2:10 PM |
| 8 | Increased dose of antegrade Custodiol + 28deg systemic temp, or sternotomy | 4/13/2022 2:07 PM |
| 9 | Direct Ostial cardioplejia through an aortotomy and also aortic valve surgery | 4/3/2022 11:30 PM |
| 10 | We use retrograde cardiopleagia | 3/31/2022 8:51 AM |
| 11 | Cardioplegia down the coronary Ostia! | 3/30/2022 3:22 PM |
| 12 | Hypothermia and fibrillation, LV venting | 3/29/2022 10:42 AM |
| 13 | Antegrade | 3/28/2022 10:54 PM |
| 14 | Hypothermic fibrillatory arrest | 3/28/2022 8:28 PM |
| 15 | Antegrade | 3/28/2022 8:02 PM |
| 16 | Retrograde cardioplegia | 3/28/2022 6:55 PM |
| 17 | Mild cooling and cold cardioplegia, rarely a retrograde | 3/28/2022 2:54 PM |
| 18 | No mimvs | 3/28/2022 12:25 PM |
| 19 | See above | 3/28/2022 10:11 AM |
| 20 | if severe is a contraindication | 3/28/2022 10:08 AM |
| 21 | with the succion lifting the back of the Aorta upward and TOE guided assessment of the level of regurgitaion | 3/28/2022 10:01 AM |
| 22 | none | 3/28/2022 8:02 AM |
| 23 | antegrade root up to moderate AI, retrograde for greater AI | 3/25/2022 1:36 PM |
| 24 | lower temperature, repeated application of cardioplegia | 3/24/2022 1:40 PM |
| 25 | sternotomy | 3/23/2022 3:54 PM |
| 26 | Increase dose of antegrade cardioplegia | 3/22/2022 9:11 PM |
| 27 | repair or change the valve | 3/22/2022 3:23 PM |
| 28 | Direct retrograde in addition to ante grade | 3/22/2022 11:36 AM |
| 29 | AR>=moderate -> change Access to partial sternotomy and retrograde cardioplegia | 3/22/2022 9:31 AM |
| 30 | hypothermia and retograde | 3/21/2022 6:27 PM |
| 31 | antegrade with direct coronary artery ostial perfusion | 3/21/2022 2:15 PM |

32 retrograde 3/21/2022 1:57 PM

# Q36 Does ascending aortic calcification preclude a patient from MIMVS?

Answered: 32 Skipped: 0

Yes

No

0% 10% 20% 30% 40% 50% 60% 70% 80% 90% 100%

| **ANSWER CHOICES RESPONSES** | |
| --- | --- |
| Yes | 31.25% 10 |

No 68.75% 22

TOTAL 32

# Q37 If no, what technique do your use to perform the MIMVS?

Answered: 23 Skipped: 9

Cross clamp

|  | | | | | |  |  |  |  |
| --- | --- | --- | --- | --- | --- | --- | --- | --- | --- |
|  |  |  |  |  |  |  |  |  |  |
|  | | |  |  |  |  |  |  |  |
|  |  |  |  |  |  |  |  |  |  |

Endo-aortic

balloon

Fibrillating heart withou...

Beating heart without cros...

Other (please

specify)

0% 10% 20% 30% 40% 50% 60% 70% 80% 90% 100%

| **ANSWER CHOICES** | **RESPONSES** | |
| --- | --- | --- |
| Cross clamp | 21.74% | 5 |
| Endo-aortic balloon | 39.13% | 9 |
| Fibrillating heart without cross clamp | 52.17% | 12 |
| Beating heart without cross clamp | 30.43% | 7 |

Other (please specify)

4.35% 1

Total Respondents: 23

| **#** | **OTHER (PLEASE SPECIFY)** | **DATE** |
| --- | --- | --- |
| 1 | Rapid pacing | 3/28/2022 10:54 PM |

# Q38 Is severe peripheral vascular disease a contraindication for MIMVS?

Answered: 32 Skipped: 0

Yes

|  | | | | | | |  |  |  |
| --- | --- | --- | --- | --- | --- | --- | --- | --- | --- |
|  | |  |  |  |  |  |  |  |  |
|  |  |  |  |  |  |  |  |  |  |

No

Other (please

specify)

0% 10% 20% 30% 40% 50% 60% 70% 80% 90% 100%

| **ANSWER CHOICES** | **RESPONSES** | |
| --- | --- | --- |
| Yes | 25.00% | 8 |
| No | 62.50% | 20 |

Other (please specify)

12.50% 4

TOTAL 32

| **#** | **OTHER (PLEASE SPECIFY)** | **DATE** |
| --- | --- | --- |
| 1 | Ok | 4/13/2022 2:10 PM |
| 2 | Alternative cannulation | 3/28/2022 10:11 AM |
| 3 | if Femoro-iliaque YES | 3/28/2022 10:01 AM |

4 relative contraindication 3/21/2022 2:15 PM

# Q39 If no, what cannulation strategy do you use? Please state.

Answered: 24 Skipped: 8

| **#** | **RESPONSES** | **DATE** |
| --- | --- | --- |
| 1 | axillary cannulation percutaneously | 4/15/2022 4:52 PM |
| 2 | Direct ascending aorta cannulation | 4/15/2022 1:41 PM |
| 3 | Axillary or direct aortic | 4/14/2022 3:38 PM |
| 4 | direct ascending or axillary a | 4/14/2022 7:36 AM |
| 5 | Bilateral femoral artery or axillary artery | 4/13/2022 5:19 PM |
| 6 | If redo or young patient, we could think of axillary cannulation | 4/13/2022 2:10 PM |
| 7 | Axillary | 4/13/2022 2:07 PM |
| 8 | Subclavian | 4/3/2022 11:32 PM |
| 9 | Axillary artery | 3/30/2022 3:22 PM |
| 10 | Axillary, or direct through working port | 3/29/2022 10:43 AM |
| 11 | Axillary | 3/28/2022 10:54 PM |
| 12 | Axillary cannulation | 3/28/2022 8:29 PM |
| 13 | Peripheral | 3/28/2022 8:02 PM |
| 14 | Axillary artery | 3/28/2022 10:11 AM |
| 15 | Enlarge a bit the thoracotomy and cannulate the ascending aorta | 3/28/2022 10:08 AM |
| 16 | axillary | 3/25/2022 1:36 PM |
| 17 | subclavian artery | 3/24/2022 1:40 PM |
| 18 | Axillary arterial cannulation | 3/22/2022 9:11 PM |
| 19 | central cannulation | 3/22/2022 3:23 PM |
| 20 | Central cannulation | 3/22/2022 11:36 AM |
| 21 | Carotid or axillary artery | 3/22/2022 9:32 AM |
| 22 | axillary arterial fem venous | 3/21/2022 6:28 PM |
| 23 | axillary | 3/21/2022 2:15 PM |

24 right axillary 3/21/2022 1:59 PM

# Q40 What degree of LV impairment is a contraindication to MIMVS?

Answered: 32 Skipped: 0

< 50%

|  | |  |  |  |  |  |  |  |  |
| --- | --- | --- | --- | --- | --- | --- | --- | --- | --- |
|  |  |  |  |  |  |  |  |  |  |
|  | | | |  |  |  |  |  |  |
|  |  |  |  |  |  |  |  |  |  |
|  | | | |  |  |  |  |  |  |
|  |  |  |  |  |  |  |  |  |  |

30 – 50%

30 – 20%

< 20%

None

Other (please

specify)

0% 10% 20% 30% 40% 50% 60% 70% 80% 90% 100%

| **ANSWER CHOICES RESPONSES** | | | |
| --- | --- | --- | --- |
| < | 50% | 0.00% | 0 |
| 30 – 50% | | 6.25% | 2 |
| 30 – 20% | | 12.50% | 4 |
| < | 20% | 40.63% | 13 |
| None | | 34.38% | 11 |

Other (please specify)

6.25% 2

TOTAL 32

| **#** | **OTHER (PLEASE SPECIFY)** | **DATE** |
| --- | --- | --- |
| 1 | This degree of LV function is too low. Consider mitral clip | 3/28/2022 8:29 PM |

2 Depends but generally < 20% where I prefer repair via sternotomy for potential temp LVAD backup

3/25/2022 1:36 PM

# Q41 Which previous surgery or intervention would preclude a patient for MIMVS?

Answered: 32 Skipped: 0

Mitral

|  |  | | | | | |  |  |  |
| --- | --- | --- | --- | --- | --- | --- | --- | --- | --- |
|  |  |  |  |  |  |  |  |  |  |
|  |  |  |  |  |  |  |  |  |  |
|  |  |  |  |  |  |  |  |  |  |
|  |  |  |  |  |  |  |  |  |  |
|  |  |  |  |  |  |  |  |  |  |
|  |  |  |  |  |  |  |  |  |  |
|  |  |  |  |  |  |  |  |  |  |
|  |  |  |  |  |  |  |  |  |  |
|  | | | | | | |  |  |  |
|  | | |  |  |  |  |  |  |  |
|  | |  |  |  |  |  |  |  |  |

TAVI

Aortic

Tricuspid

CABG

Ipsilateral Thoracic...

Chest radiotherapy

All Cardiac Surgery

Chest trauma

None Other (please

specify)

0% 10% 20% 30% 40% 50% 60% 70% 80% 90% 100%

| **ANSWER CHOICES** | **RESPONSES** | |
| --- | --- | --- |
| Mitral | 6.25% | 2 |
| TAVI | 9.38% | 3 |
| Aortic | 9.38% | 3 |
| Tricuspid | 9.38% | 3 |
| CABG | 9.38% | 3 |
| Ipsilateral Thoracic surgery | 62.50% | 20 |
| Chest radiotherapy | 25.00% | 8 |
| All Cardiac Surgery | 3.13% | 1 |
| Chest trauma | 18.75% | 6 |
| None | 25.00% | 8 |

Other (please specify)

15.63% 5

Total Respondents: 32

| **#** | **OTHER (PLEASE SPECIFY)** | **DATE** |
| --- | --- | --- |
| 1 | But are relative contraindications | 4/3/2022 11:32 PM |
| 2 | In general, ipsilateral thoracic surgery. But we have attempted and performed all kinds of borderline cases | 3/29/2022 10:43 AM |
| 3 | generally, none depends on the right sided surgery - if pneumonectomy, then yes, but other  less thoracic surgery are still candidates | 3/25/2022 1:36 PM |
| 4 | expected adhesions (opened pleural space) | 3/24/2022 1:40 PM |

5 right sided lung recestion 3/23/2022 3:54 PM

# Q42 Do you consider mitral annular calcification (MAC) as a contraindication to MIMVS?

Answered: 32 Skipped: 0

Yes

|  | | | | | |  |  |  |  |
| --- | --- | --- | --- | --- | --- | --- | --- | --- | --- |
|  |  |  |  |  |  |  |  |  |  |
|  |  |  |  |  |  |  |  |  |  |
|  |  |  |  |  |  |  |  |  |  |

No

Other (please

specify)

0% 10% 20% 30% 40% 50% 60% 70% 80% 90% 100%

| **ANSWER CHOICES** | **RESPONSES** | |
| --- | --- | --- |
| Yes | 34.38% | 11 |
| No | 56.25% | 18 |

Other (please specify)

9.38% 3

TOTAL 32

| **#** | **OTHER (PLEASE SPECIFY)** | **DATE** |
| --- | --- | --- |
| 1 | Yes, if severe | 4/13/2022 5:19 PM |
| 2 | Only if severe but have done a lot | 3/28/2022 8:29 PM |

3 extensive MAC yes 3/23/2022 3:54 PM

# Q43 If no, what is your approach to MAC in MIMVS?

Answered: 19 Skipped: 13

De-calcifying the annulus

|  | | | | | | |  |  |  |
| --- | --- | --- | --- | --- | --- | --- | --- | --- | --- |
|  | | | | |  |  |  |  |  |
|  |  |  |  |  |  |  |  |  |  |

Open trans-cathet...

Other

Other (please

specify)

0% 10% 20% 30% 40% 50% 60% 70% 80% 90% 100%

| **ANSWER CHOICES** | **RESPONSES** | |
| --- | --- | --- |
| De-calcifying the annulus | 63.16% | 12 |
| Open trans-catheter valve implantation | 42.11% | 8 |
| Other | 0.00% | 0 |

Other (please specify)

47.37% 9

Total Respondents: 19

| **#** | **OTHER (PLEASE SPECIFY)** | **DATE** |
| --- | --- | --- |
| 1 | Don’t implanting any sutured anular device, if is possibile | 4/15/2022 1:41 PM |
| 2 | It depends of MAC degree. | 4/3/2022 11:32 PM |
| 3 | Suture valve into the mobile leaflets if possible. | 3/30/2022 3:22 PM |
| 4 | Case by case. Team discussion. | 3/29/2022 10:43 AM |
| 5 | Have done both | 3/28/2022 8:29 PM |
| 6 | Depends from patient. Decalcification for young and no decalcification for older | 3/28/2022 2:54 PM |
| 7 | use all options... | 3/25/2022 1:36 PM |
| 8 | atrial implantation with a pericardial skirt | 3/24/2022 1:40 PM |

9 replacement and leave as much calcium as possible 3/23/2022 3:54 PM

# Q44 Do you consider mitral valve endocarditis as a contraindication to MIMVS?

Answered: 32 Skipped: 0

Yes

|  | | | | | | | | |  |
| --- | --- | --- | --- | --- | --- | --- | --- | --- | --- |
|  |  |  |  |  |  |  |  |  |  |
|  |  |  |  |  |  |  |  |  |  |
|  |  |  |  |  |  |  |  |  |  |

No

Other (please

specify)

0% 10% 20% 30% 40% 50% 60% 70% 80% 90% 100%

| **ANSWER CHOICES** | **RESPONSES** | |
| --- | --- | --- |
| Yes | 6.25% | 2 |
| No | 84.38% | 27 |

Other (please specify)

9.38% 3

TOTAL 32

| **#** | **OTHER (PLEASE SPECIFY)** | **DATE** |
| --- | --- | --- |
| 1 | acute (hot) endocarditis should be done open. | 4/14/2022 3:38 PM |
| 2 | Not unless there is an annular abscess | 4/13/2022 2:07 PM |

3 Only in very extended pathology 3/22/2022 9:32 AM

# Q45 Which of the following concomitant procedures in your practice are contraindication to MIMVS?

Answered: 29 Skipped: 3

Concomitant Tricuspid

Concomitant

Maze

Concomitant Tricuspid an...

Septal myectomy

None

Other (please

specify)

0% 10% 20% 30% 40% 50% 60% 70% 80% 90% 100%

| **ANSWER CHOICES** | **RESPONSES** | |
| --- | --- | --- |
| Concomitant Tricuspid | 10.34% | 3 |
| Concomitant Maze | 17.24% | 5 |
| Concomitant Tricuspid and Maze | 20.69% | 6 |
| Septal myectomy | 37.93% | 11 |
| None | 55.17% | 16 |

Other (please specify)

0.00% 0

Total Respondents: 29

**# OTHER (PLEASE SPECIFY) DATE**

There are no responses.

# Q46 What are the indications for conversion to a full sternotomy in your MIMVS practice?

Answered: 29 Skipped: 3

Lung adhesions

|  | | | | | | |  |  |  |
| --- | --- | --- | --- | --- | --- | --- | --- | --- | --- |
|  |  |  |  |  |  |  |  |  |  |
|  | |  |  |  |  |  |  |  |  |
|  |  |  |  |  |  |  |  |  |  |
|  | |  |  |  |  |  |  |  |  |
|  |  |  |  |  |  |  |  |  |  |
|  | |  |  |  |  |  |  |  |  |
|  |  |  |  |  |  |  |  |  |  |
|  | | |  |  |  |  |  |  |  |

Poor Exposure of mitral valve

Complexity of mitral valve...

Inadequate drainage

Revision of repair durin...

Diﬃculty to use endo-aor...

Diﬃculty to place aortic...

None Other (please

specify)

0% 10% 20% 30% 40% 50% 60% 70% 80% 90% 100%

| **ANSWER CHOICES** | **RESPONSES** | |
| --- | --- | --- |
| Lung adhesions | 65.52% | 19 |
| Poor Exposure of mitral valve | 20.69% | 6 |
| Complexity of mitral valve pathology | 3.45% | 1 |
| Inadequate drainage | 17.24% | 5 |
| Revision of repair during same surgery | 0.00% | 0 |
| Difficulty to use endo-aortic balloon | 0.00% | 0 |
| Difficulty to place aortic cross clamp | 6.90% | 2 |
| None | 20.69% | 6 |

Other (please specify)

27.59% 8

Total Respondents: 29

**# OTHER (PLEASE SPECIFY) DATE**

| 1 | Uncontrollable Profuse bleeding | 4/15/2022 1:41 PM |
| --- | --- | --- |
| 2 | Severe hemorrhage, which cannot be salvaged through workport | 3/29/2022 10:43 AM |
| 3 | intractable bleeding | 3/28/2022 10:08 AM |
| 4 | for all above , I try to overcome first but keeping a low threshold for conversion as safety Did not have any so far | 3/28/2022 10:01 AM |
| 5 | intraoperative catastrophe, aortic dissection, uncontrollable bleeding, AV groove disruption | 3/25/2022 1:36 PM |
| 6 | if severe and hard to solve | 3/24/2022 1:40 PM |
| 7 | LV bleeding | 3/23/2022 3:54 PM |

8 bleeding 3/22/2022 3:23 PM

# Q47 In your opinion, surgeons in the early stage of MIMVS encountering the above complexities, should they convert to a full sternotomy early?

Answered: 32 Skipped: 0

Yes

|  | | | | | | | |  |  |
| --- | --- | --- | --- | --- | --- | --- | --- | --- | --- |
|  | |  |  |  |  |  |  |  |  |
|  |  |  |  |  |  |  |  |  |  |

No

Other (please

specify)

0% 10% 20% 30% 40% 50% 60% 70% 80% 90% 100%

| **ANSWER CHOICES** | **RESPONSES** | |
| --- | --- | --- |
| Yes | 75.00% | 24 |
| No | 6.25% | 2 |

Other (please specify)

18.75% 6

TOTAL 32

| **# OTHER (PLEASE SPECIFY) DATE** | | |
| --- | --- | --- |
| 1 | using metor / mentee concept | 3/24/2022 1:40 PM |
| 2 | they should ask supervisor. | 3/23/2022 3:54 PM |
| 3 | make larger incision and use the retractor | 3/22/2022 3:23 PM |
| 4 | depends on the issue | 3/21/2022 6:28 PM |
| 5 | Depends on the experience of the surgeon. The goal would not to do the case in the first place with good preoperative imaging and planning if felt to be at high risk for conversion | 3/21/2022 2:15 PM |

6 surgical stepwise team-approach with smooth transition expert > beginner MIC surgeon 3/21/2022 2:00 PM

# Q48 What is your deairing strategy for MIMVS?

Answered: 32 Skipped: 0

| **# RESPONSES DATE** | | |
| --- | --- | --- |
| 1 | Refilling with saline and massage prior to release cross clamp, needlevent in aortic root | 5/2/2022 5:35 AM |
| 2 | CO2, leave volume in with root vent on prior to balloon deflation, reverse trendelenberg position (head up) | 4/15/2022 4:52 PM |
| 3 | The same of sternotomy plus CO2 ambient saturation | 4/15/2022 1:41 PM |
| 4 | Via the root, firstly with the head up and then head down prior to de-clamping. Ventilating the left lung while de-airing. | 4/14/2022 3:38 PM |
| 5 | CO2 insuflation in thorax and after procedure | 4/14/2022 7:36 AM |
| 6 | CO2, retrograde and antegrade deairing, tilting the table ad external cardiac massage | 4/13/2022 5:19 PM |
| 7 | From aortic root and LA incision | 4/13/2022 2:10 PM |
| 8 | Deairing across the LA suture line, leaving an LV vent in situ Deairing of LV and root through root and Lv vent Unclamping | 4/13/2022 2:07 PM |
| 9 | CO2 in chest cavity and root deairing after aortic clamp removal under eco guidance | 4/3/2022 11:32 PM |
| 10 | We use CO2 in operating field and pediatric defibrillating pads to compress LV apex and antegrade root vent . | 3/31/2022 8:52 AM |
| 11 | Endoclamp does the deairing. | 3/30/2022 3:22 PM |
| 12 | CO2 and avoiding too much shaking (Coca-Cola effect) | 3/29/2022 10:43 AM |
| 13 | LV and Ao root venting | 3/28/2022 10:54 PM |
| 14 | Co2 through the endoscopic port during the entire case. Cannulation of the ascending aorta with active withdrawal from vent when coming off bypass | 3/28/2022 8:29 PM |
| 15 | CO2 and filling of left atrium before closure, then use of root vent. | 3/28/2022 8:02 PM |
| 16 | I do not. Use CO2 instead | 3/28/2022 6:55 PM |
| 17 | Use of co2 and fill the heart during atrial closure and left side ventilation. | 3/28/2022 2:54 PM |
| 18 | CO2 aspiration on aorta | 3/28/2022 12:26 PM |
| 19 | CO2 Insufflation, venous uploading venting through ascending aorta | 3/28/2022 10:12 AM |
| 20 | CO2 flooding | 3/28/2022 10:08 AM |
| 21 | through LA incision and Aortic root | 3/28/2022 10:01 AM |
| 22 | CO2 and TEE | 3/28/2022 8:03 AM |
| 23 | post-repair, fill the ventricle with as much saline as possible, rotate bed, valsalva and after LA closed, vent the air out the root vent also CO2 insufflation throughout the entire case | 3/25/2022 1:36 PM |
| 24 | fill atria while suturing venting via balloon / cardioplegia needle | 3/24/2022 1:40 PM |
| 25 | CO2 an fill the heart and ventilate with left lung | 3/23/2022 3:54 PM |
| 26 | CO2 infusion with progressive filling of the heart before closure of the left atrium, followed by inflation of the lungs and starting the aortic vent | 3/22/2022 9:11 PM |
| 27 | vent and CO2 | 3/22/2022 3:23 PM |
| 28 | CO2 insufflation aortic vent | 3/22/2022 11:37 AM |

29 Ascending aorta using the cardioplegia line 3/22/2022 9:32 AM

| 30 | CO2 insufflation , deairing prior to closure , aortic vent | 3/21/2022 6:28 PM |
| --- | --- | --- |
| 31 | Routine | 3/21/2022 2:15 PM |

32 CO2 3/21/2022 2:00 PM

# Q49 For those starting with MIMVS, what would you recommend regarding the conditioning?

Answered: 29 Skipped: 3

Hypothermia and crystall...

|  | | | | | | |  |  |  |
| --- | --- | --- | --- | --- | --- | --- | --- | --- | --- |
|  | |  |  |  |  |  |  |  |  |
|  |  |  |  |  |  |  |  |  |  |

Start with external cla...

Other (please

specify)

0% 10% 20% 30% 40% 50% 60% 70% 80% 90% 100%

| **ANSWER CHOICES** | **RESPONSES** | |
| --- | --- | --- |
| Hypothermia and crystalloid cardioplegia | 48.28% | 14 |
| Start with external clamp and never directly with intra-aortic balloon | 68.97% | 20 |

Other (please specify)

13.79% 4

Total Respondents: 29

| **# OTHER (PLEASE SPECIFY) DATE** | | |
| --- | --- | --- |
| 1 | Use cardioplegia you normally use and chitwood is a good starting point. | 4/14/2022 3:38 PM |
| 2 | Endoclamp after a fellowship. | 3/30/2022 3:22 PM |
| 3 | Not sure I understand the question. Yes, can go colder, but would still recommend del nido, and clamp or endoballoon, depending on what they feel more comfortable with | 3/25/2022 1:36 PM |

4 Start with a technique you are familiar with 3/22/2022 9:11 PM

# Q50 For those starting with MIMVS, what would you recommend regarding the approach?

Answered: 29 Skipped: 3

First direct-visio...

|  | | | | | | | | |  |
| --- | --- | --- | --- | --- | --- | --- | --- | --- | --- |
|  | |  |  |  |  |  |  |  |  |
|  |  |  |  |  |  |  |  |  |  |

From beginning

at least...

Other (please

specify)

0% 10% 20% 30% 40% 50% 60% 70% 80% 90% 100%

| **ANSWER CHOICES** | **RESPONSES** | |
| --- | --- | --- |
| First direct-vision and gradually convert to endoscopic | 86.21% | 25 |
| From beginning at least endoscopic approach to the valve | 17.24% | 5 |

Other (please specify)

6.90% 2

Total Respondents: 29

| **#** | **OTHER (PLEASE SPECIFY)** | **DATE** |
| --- | --- | --- |
| 1 | Endoscopic vision is never necessary | 4/15/2022 1:41 PM |

2 depends on the individual 3/25/2022 1:36 PM

# Q51 For those starting with MIMVS, which of the following patients’ categories should be excluded:

Answered: 32 Skipped: 0

Peripheral arterial...

|  | | | | | | | | | |
| --- | --- | --- | --- | --- | --- | --- | --- | --- | --- |
|  | | | | | | |  |  |  |
|  | | | | |  |  |  |  |  |
|  |  |  |  |  |  |  |  |  |  |
|  | | | | |  |  |  |  |  |
|  |  |  |  |  |  |  |  |  |  |

AR

Complex mitral

valve...

Chest deformity

Concomitant procedures

Moderate and poor left...

Right ventricle...

Redo-operation

Endocarditis

Octogenarians

PHT

Other (please

specify)

0% 10% 20% 30% 40% 50% 60% 70% 80% 90% 100%

| **ANSWER CHOICES** | **RESPONSES** | |
| --- | --- | --- |
| Peripheral arterial disease | 68.75% | 22 |
| AR | 68.75% | 22 |
| Complex mitral valve pathology, other than P2 prolapse | 78.13% | 25 |
| Chest deformity | 84.38% | 27 |
| Concomitant procedures | 71.88% | 23 |
| Moderate and poor left ventricle function | 78.13% | 25 |
| Right ventricle dysfunction | 87.50% | 28 |

Redo-operation

93.75% 30

| Endocarditis | 62.50% 20 |
| --- | --- |
| Octogenarians | 43.75% 14 |
| PHT | 50.00% 16 |

Other (please specify)

6.25% 2

Total Respondents: 32

| **#** | **OTHER (PLEASE SPECIFY)** | **DATE** |
| --- | --- | --- |
| 1 | Exclusion criteria depends on the feasibility of operating centre to approach and manage the patient depending upon there complexity. | 3/31/2022 8:52 AM |

2 Maybe obese 3/28/2022 8:29 PM

# Q52 Any suggestions to improve future surveys?

Answered: 19 Skipped: 13

| **#** | **RESPONSES** | **DATE** |
| --- | --- | --- |
| 1 | have a experience surgeon for the first 50 cases close by | 5/2/2022 5:35 AM |
| 2 | Coose a good Teacher | 4/15/2022 1:41 PM |
| 3 | No | 4/13/2022 2:10 PM |
| 4 | No | 4/13/2022 2:07 PM |
| 5 | No | 4/3/2022 11:32 PM |
| 6 | 1. Idea of young surgeons should be included. 2. Prepare certain guidlines for all young surgeons to follow universally | 3/31/2022 8:52 AM |
| 7 | No | 3/30/2022 3:22 PM |
| 8 | We can contribute a lot of questions, from our 10 year proctorship and setting -up programs in Asia - experience | 3/29/2022 10:43 AM |
| 9 | This is excellent! | 3/28/2022 8:29 PM |
| 10 | Echocardiographic assessment of the valve prior surgery | 3/28/2022 10:12 AM |
| 11 | Strategy of aortic occlusion ; Double or single venous cannulation; selective lung ventilation or not; | 3/28/2022 8:03 AM |
| 12 | Good survey. Would be happy to participate in the design of future surveys/studies and would also be interested in collaborating in analyzing and writing up this study if there was interest | 3/25/2022 1:36 PM |
| 13 | no, thanx | 3/24/2022 1:40 PM |
| 14 | Best regards, | 3/23/2022 3:54 PM |
| 15 | Some answers are difficult because it will depend on many patients factors | 3/22/2022 9:11 PM |
| 16 | none | 3/22/2022 3:23 PM |
| 17 | many of the questions needed to have more than one choice pickable and only one or two could actuallyn allow | 3/21/2022 6:28 PM |
| 18 | The following may be of assistance: J Thorac Cardiovasc Surg. 2021 Nov 10:S0022- 5223(21)01541-5. doi: 10.1016/j.jtcvs.2021.11.005. Online ahead of print. | 3/21/2022 2:15 PM |

19 no 3/21/2022 2:00 PM
